# Sex differences in microRNA regulation of gene expression: no smoke, just miRs

**DOI:** 10.1186/2042-6410-3-22

**Published:** 2012-09-26

**Authors:** Christopher P Morgan, Tracy L Bale

**Affiliations:** 1 Department of Animal Biology, School of Veterinary Medicine, University of Pennsylvania, 3800 Spruce Street, Ste. 201E, Philadelphia, PA, 19104-6046, USA

**Keywords:** MicroRNA, Sex difference, Sex-bias, Sexual differentiation, Post-transcriptional regulation

## Abstract

Males and females differ widely in morphology, physiology, and behavior leading to disparities in many health outcomes, including sex biases in the prevalence of many neurodevelopmental disorders. However, with the exception of a relatively small number of genes on the Y chromosome, males and females share a common genome. Therefore, sexual differentiation must in large part be a product of the sex biased expression of this shared genetic substrate. microRNAs (miRs) are small non-coding RNAs involved in the post-transcriptional regulation of up to 70% of protein-coding genes. The ability of miRs to regulate such a vast amount of the genome with a high degree of specificity makes them perfectly poised to play a critical role in programming of the sexually dimorphic brain. This review describes those characteristics of miRs that make them particularly amenable to this task, and examines the influences of both the sex chromosome complement as well as gonadal hormones on their regulation. Exploring miRs in the context of sex differences in disease, particularly in sex-biased neurodevelopmental disorders, may provide novel insight into the pathophysiology and potential therapeutic targets in disease treatment and prevention.

## Review

### Introduction

A decade of genome-wide association studies has identified numerous genetic loci associated with neurodevelopmental disorder susceptibility. While statistically significant, the effect size of individual loci has tended to be quite small. In contrast, the presence of either a Y or a second X chromosome is likely one of the strongest genetic predictors of many aspects of neurodevelopmental disorders, including prevalence, presentation, and therapeutic outcome. Sex differences in health outcomes are not limited to neurodevelopmental disease; instead, the importance of sex has been highlighted in immune-related diseases, many cancers, and coronary heart disease as examples [1-3]. Recognition of these sex-biases in disease is leading to investigation of the underlying molecular processes responsible for sexual differentiation of cells and tissues. 

**Table 1:** Common molecules in microRNA biogenesis and function

**Molecule**	**Function**	**Reference**
Primary microRNA (pri-miR)‘	Initial transcription from independent (not intronic) miR gene results in a pri-miR, A pri-miR is often multiple kilobases in length and may encode more then one mature miRNA.	[12]
Pre-microRNA (pre-miR)	Processing of pri-miR by microprocessor results in pre-miR. PremiRs are 60–70 bp double stranded RNAs with intramolecular sequence complementarity, so that they form stem-loop structures.	[20]
microRNA (miR)	Mature single stranded RNA 20–22 bp in length. Exists in complex w ith Argonaute and accessory proteins as part of the RISC complex, providing target specificity.	[20]
Mirtron	miRs located within introns of protein coding genes, Transcribed as part of their host genes primary transcript, but are cleaved from this during mRNA splicing, resulting in a pre-miR.	[15]
Microprocessor complex	Multi-protein complex, containing the obligate members Drosha and DGCR8. Responsible for cleaving stem-loop structures from pn-miR, resulting in pre-miRNA.	[52]
Drosha	RNase III protein. One of two obligate members of the microprocessor complex.	[73]
DGCR8	Double stranded RNA binding domain protein. One of two obligate members of the microprocessor complex.	[73]
Dicer	RNase Ill-like protein. Cleaves ‘loops of stem-loop from pre-miR, resulting in a 20–22 bp miRNA duplex. Assists in loading one strand of this duplex, the guide strand, into RISC complex.	[12]
RISC	RNA induced silencing complex (RISC) is a multi-protein complex containing Argo bound to a single stranded miR. Association of the RISC complex with a mRNA target leads to mRNAdestabilization/degradation, either through direct RNase actions of Argo II or the activity of other recruited accessory proteins.	[74]
Argonaute (Argo)	Component of the RISC complex, it acts at the interface between a miR and mRNA target. There are four mammalian Argonautes, though only Argo II has RNase activity.	[16][74]

It is becoming clear that the complexity of gene expression responses, or the conversion of information contained in a genetic element into a molecular effector, results from regulatory mechanisms beyond the simple interactions of transcription factors and DNA cis-regulatory sequences. Instead, appropriate regulation of gene expression results from a controlled balance between transcriptional and post-transcriptional mechanisms. With this in mind, we have begun to examine microRNAs (miRs) in the context of sex differences. miRs are essential for both the development and adult function of all tissues, and the dysregulation of miR expression has been linked to a variety of human diseases, such as cancer, immune dysfunction, metabolic, and cardiovascular diseases [2,4-7]. Recent studies have begun identifying sex differences and specificity in miR responses to pathologic conditions, such as cerebral ischemia and radiation, and in cancers including hepatocellular and squamous cell carcinomas [1,8-10]. In addition, recent work in our own lab has implicated the developmental miR environment in the sexual differentiation of the brain [11]. As such, the disruption of normal miR expression patterns could be a potential risk factor in neurodevelopmental disease, contributing to the sex-biases found in these disorders. In this review, we will highlight characteristics of miRs that make them particularly amenable to regulating sexual differentiation of the brain, and discuss mechanisms through which sex biased miR expression can occur.

### microRNA: biogenesis and activity

**Figure 1:**
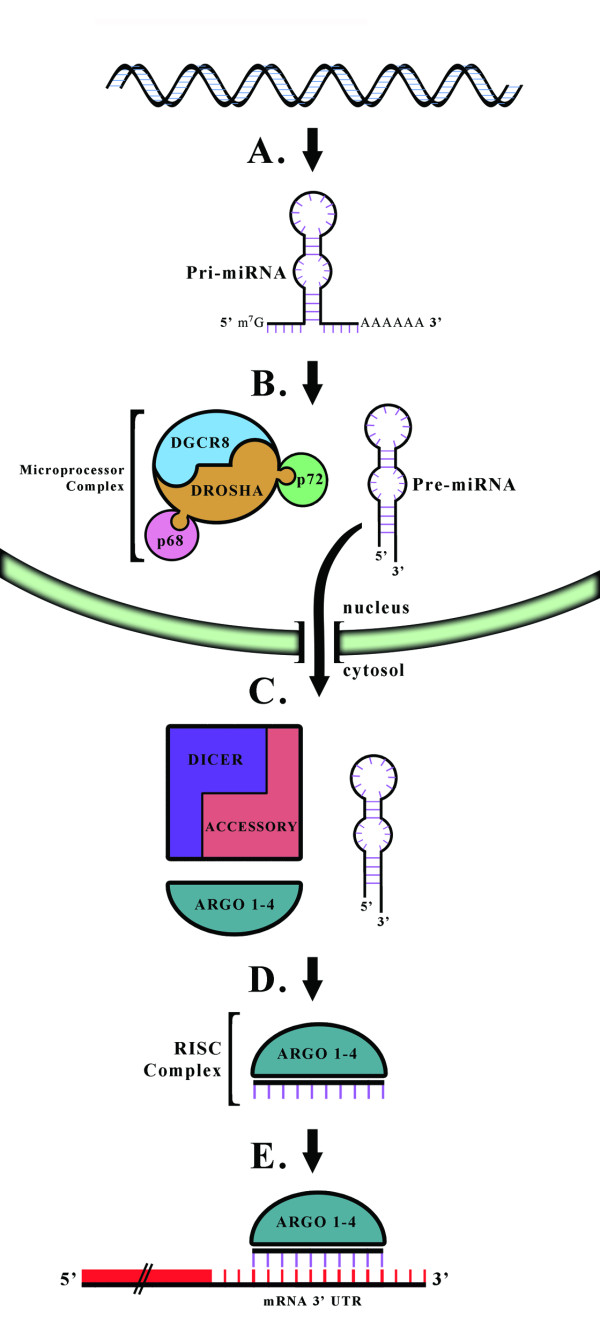
**Schematic representation of miRNA (miR) biogenesis. A**, Most miRs are transcribed as polycistronic primary-miRs (pri-miRs) by RNA polymerase II, before undergoing 5' capping and 3' polyadenylation. Portions of the pri-miR folds back on itself to form double-stranded stem-loop structures. **B**, The Microprocessor complex cleaves these stem-loops from pri-miRs, generating 50–70 bp pre-miRs with a short 3' overhang. Drosha and DGCR8 are the obligate components of the Microprocessor complex, though additional accessory proteins, such as the RNA helicases P68 and P72, can regulate the activity of the complex. **C**, The short 3' overhang is recognized by components of the nuclear export machinery, leading to active transport of pre-miRs out into the cytosol. In the cytosol, Dicer acts in complex with accessory proteins to process the pre-miRs into double stranded 22 bp duplexes. **D**, Dicer then assists in loading one strand of this duplex, the guide strand, into the Argonaute-containing RISC complex. **E**, Mature miRs guide the RISC complex to specific mRNA targets. miRs identify mRNA targets through regions of sequence homology in the mRNA's 3' UTR. The outcome of this interaction can depend on the degree of sequence complementarity and the specific Argonaute present in the RISC complex, but destabilization of the mRNA and subsequent degradation is likely.

miRs are small non-coding RNAs that regulate post-transcriptional gene expression by affecting the stability or translational efficiency of specific mRNA targets. The majority of miRs are organized in clusters within the genome, and are co-transcribed as single long poly-cistronic primary transcripts (pri-miRs) that are multiple kilobases in length (see Table 1) [12]. The transcription of these pri-miRs is RNA polymerase II dependent, and regulated by the same mechanisms as mRNAs [13]. Also similar to mRNAs, pri-miRs undergo 5’ capping and 3’ polyadenylation [14]. Portions of the pri-miR folds back on itself to form a distinct stem-loop structure (see Figure 1). In the nucleus, a Drosha-containing microprocessor cleaves the pri-miR at the base of this ‘stem’, generating a 60–70 bp precursor (pre)-miR [12]. Other miRs, termed mirtrons, are located within introns of mRNAs, and are co-transcribed with their host gene [13,15]. Mirtrons bypass Drosha processing, and instead use mRNA splicing machinery to generate pre-miRs [15]. In the cytosol, Dicer processes pre-miRs into double stranded 22 bp miR duplexes, and assists in loading one of the strands of a duplex (the guide strand) into the Argonaute-containing RNA-induced silencing complex (known as the RISC complex). Argonaute proteins act at the interface between miRs and their target mRNAs to mediate the functional consequences of these interactions [16]. There are four different Argonaute proteins found in mammals, Argo 1–4, though 60% of miR-associated RISC complexes contain Argo 2, the only Argonaute with endonuclease activity [16,17]. Mature miRs guide the RISC complex to the 3’ UTR of mRNAs, providing target specificity through partial sequence homology, and typically resulting in mRNA destabilization and degradation [18-20]. Thus, a typical rule is that with an increase in miR expression, you see a concordant decrease in the target mRNA. 

miRs act as major components of an integrated gene expression regulatory mechanism [21]. One genome-wide bioinformatics study annotated more than 45,000 conserved miR binding sites in the 3’ UTR of 60% of human genes [22]. In addition, each miR can directly target more than a hundred different mRNA targets, making this mode of regulation far-reaching and capable of profound programmatic effects [23,24]. For example, more than 600 distinct mRNA targets were identified by Argonaute immunoprecipitation following miR-124 overexpression in MCF-7 cells, an immortalized breast cancer cell line [25]. Interestingly, the presence of a miR does not necessarily result in the complete absence of expression of target transcripts. In fact, in two separate proteomic studies the transfection or deletion of a single miR (including miR-1, miR-16, miR-30a, miR-155, miR-124, miR223, or Let-7b) affected the production of hundreds of proteins. However, the magnitude of these effects on individual proteins was modest (1–2 fold changes) [23,24]. These data support a model of miR function proposed by Bartel and Chen, in which they described a functional group of miR targets, termed “tuning targets” that have taken advantage of the miR environment and machinery to develop an additional level of regulation [26]. This provides a cell with a greater ability to fine-tune gene expression in response to a continuously changing environment.

### Importance of modulators of gene expression in sexual differentiation

Males and females differ widely in morphology, physiology, and behavior. These differences are the inevitable product of natural selection acting on a sexually reproducing species. At the most basic level, eggs ‘cost’ more than sperm. Because of this fundamental sex difference in parental investment, males and females are under sex-specific selection pressures, and so are driven to develop phenotypic sex differences [27]. While the polygenic nature of most phenotypes complicates the direct linkage of sexually dimorphic traits to specific genes, these phenotypes must have a genetic underpinning. Yet, with the exception of the Y chromosome, male and female genomes do not vary in sequence, and sexual antagonism may arise in which the expression of a shared gene is adaptive in one sex, but maladaptive in the other [28]. This differential selection pressure in the context of a shared genetic substrate will drive the development of sex-specific gene expression modifiers [29,30]. miRs likely play an important, but currently underappreciated role in these regulatory processes.

Sexual selection acts foremost on the gonad/gametogenesis and the development of sex-specific behaviors, which require differentiation of the brain. Sexual differentiation of neural tissue is the result of the combined influences of genetic and hormonal differences between males and females [31,32]. Genetic differences, in the form of sex chromosome complement, are present from the time of fertilization, and influence development throughout life. In addition, the developmental trajectory and adult function of sufficiently sensitive neurons are affected by differences in sex steroid levels produced by either the testes or ovaries during specific early windows of sensitivity, as well as after sexual maturity is reached [33]. A great deal of divergent development must result from this interaction between genetic sex and the hormonal environment at the cellular level to produce a sex-specific phenotype. Cellular behavior results from the summation of all the molecular interactions occurring within the cell, including such events as receptor-ligand binding at the cell surface and the recruitment of transcriptional machinery to regions of permissive chromatin structure. Arnold and Lusis highlight the utility of modeling sex-specific cell function as the output of a given network [34]. The nodes and connections comprising this network consist of gene products and their interactions. The relative importance of a particular node would be dependent on the number of other nodes it interacts with and the strength of these interactions. Some of these nodes would be sex-biased or sex-specific, and the sum of the interactions between these nodes and their connections, ‘the sexome’, would generate sex-differences in the output of the network [34]. Taken together, the ability of miRs to target many transcripts and modulate their expression with a high degree of control and specificity suggests that they could serve as major nodes within a cell network mediating sex-specific developmental processes and functions. Of course, for sex differences to exist at the level of miRs, regulation of these molecules by gonadal hormones or sex chromosomes must exist.

### Gonadal hormone regulation of miRs

Initial studies characterizing the impact of gonadal steroids, including dihydrotestosterone, progesterone, and estradiol on miR expression patterns have generally involved the analyses of steady-state mature miR levels, often in hormone-responsive tumor samples [35-42]. While appropriate first steps, these studies were generally unable to determine *how* the hormonal change affected miR expression, and it has become clear that these mechanisms are likely quite diverse.

*Transcription effects of gonadal hormones*. Gonadal hormones acting through nuclear hormone receptors (NHR) can have direct or indirect effects on gene expression. At the most basic level, ligand-bound NHRs can directly bind to response elements in gene promoters and recruit co-activators or co-repressors to alter the activity of the transcriptional machinery [43]. As miRs are themselves genes, they too can be regulated in this manner. For example, estrogen receptor alpha (ERα) binds regulatory regions upstream of both miR-21 and -23a, stimulating their transcription in breast cancer cells [42]. It is important to keep in mind that none of these changes occur in isolation. NHRs and miRs are part of the molecular network of the cell, and secondary/downstream events can be difficult to untangle from the primary effects of the hormone once the system is assayed [44]. For example, estradiol stimulates the expression of a cluster of 6 miRs as part of the primary transcript pri-miR-17-92 in MCF-7 cells [45]. However, there is no estrogen-receptor binding element (ERE) upstream of pri-miR-17-92. Instead, the induction of this transcript is dependent on the activity of the ERα target gene c-Myc. ERα activates c-Myc transcription by binding an upstream ERE, which in its role as a transcription factor binds the miR-17-92 promoter and stimulates its transcription [45]. Hence, similar to other genes, miRs can be primary or secondary transcriptional targets of gonadal hormones.

While it is always the case that the responses of cancer cells may not reflect those of normal tissues, recent studies of miRs in estrogen-responsive breast cancers have begun to shed light on the dynamics of NHR activation of miR genes. A 2011 study by Hah *et al.* provided a thorough analysis of the effects of sex steroid signaling on the transcriptome [21]. This group performed a global run-on assay in cells cultured with estradiol for acute time courses (10–40 min). This technique allowed them to identify only those primary transcripts that were being actively transcribed, and so were likely primary targets of an estrogen receptor. They identified 322 distinct pri-miRs in their dataset. Of these, 37% were regulated by estradiol [21]. As they had observed in protein-coding transcripts, a nearly equal number of miR-containing transcripts were induced as were suppressed by estradiol [21]. Interestingly, they found that nearly 20% of estradiol-regulated mRNAs that were predicted targets of miRs also identified as being regulated by estrogen in this assay. Surprisingly, the direction of the estradiol effect on a given miR did not predict whether its potential mRNA target was correspondingly induced or repressed. Thus, the interaction between estradiol-responsive miRs and mRNA transcription may be coordinated (miR suppressed while its target mRNA is induced) or compensatory (both miR and its mRNA target induced). These findings highlight the extent to which miR post-transcriptional actions are integrated into a broader gene regulatory machinery that is responsive to gonadal hormones [21].

*Post-transcriptional effects of gonadal hormones.* In addition to transcriptional effects, it is becoming clear that some NHRs directly interact with components of the miR processing machinery, influencing miR processing [46]. Similar to mRNAs, miRs are regulated at seemingly every step of their post-transcriptional processing, and instances of estradiol acting at these points have been documented [47,48]. Drosha and Dicer act as components of larger complexes containing accessory proteins, often double-stranded RNA-binding proteins, which can activate or repress the activity of these complexes. These actions may be specific to individual miRs or families of miRs, or broadly affect processing of all of the miRs expressed by the cell [49-51]. Examples of hormonal regulation of miR processing are growing in number. Yamagata *et al.* performed a microarray analysis of uterine tissue samples from ovariectomized female mice treated with estradiol, and identified 39 miRs that were suppressed, though surprisingly none that were induced [52]. A subset of these miRs was further examined by northern blot analysis, and while the reduced levels of mature miR were confirmed, no effect of estradiol on precursor pri-miR levels was observed [52]. These data suggested that estradiol was affecting the post-transcriptional processing of these miRs. Subsequent experiments demonstrated that ligand-bound ERα inhibits the processing of many estrogen regulated miRs by directly interacting with a portion of Drosha, effectively blocking its nuclease and RNA-binding activities. This interaction was greatly strengthened by the presence of two accessory proteins of the Drosha-containing microprocessor complex, P68 and P72 [52]. Importantly, not all of the miRs affected by estradiol required this P68/72-dependent interaction, supporting a role for these and other cofactors in providing specificity to the pools of miRs regulated by a particular signal [53]. In addition, Dicer and Argonaute gene expression is also responsive to estrogenic signaling in breast cancer cell lines [42,54]. The effect on Dicer expression was associated with ERα binding to an identified ERE approximately 40 kb downstream of this gene [42,54]. In summay, these studies illustrate that gonadal hormones can affect mature miR levels, both directly and indirectly, through canonical NHR activity at the transcriptional level, as well as by regulating miR post-transcriptional processing, producing broad-reaching programmatic effects within cells and across tissues.

### Sex-chromosomal regulation of miRs

Sex chromosomes typically generate sex-biased gene expression for two primary reasons: 1) Genes located on the Y chromosome can only be expressed in males, and 2) Female cells contain two copies of the X chromosome, while males only have one. As the Y chromosome contains no miR genes, hormone-independent mechanisms of sex-biased miR regulation are likely restricted to those miRs that are encoded on the X chromosome or are regulated by X-linked factors [55]. The mammalian X chromosome is highly enriched for miRs [56]. The density of miRs on the X chromosome is approximately two-fold higher than on autosomes in mice and humans [56]. The X chromosome is also enriched for genes with sex-biased expression involved in behavior and sexual differentiation of many tissues, including the gonad [57,58]. This is due to the fact that the X chromosome experiences sex-specific selection pressure [59]. For example, when dominant alleles that benefit females at the cost of males are located on the X chromosome, they are only exposed to negative selection pressure in males one-third of the time, as one of the three states the X chromosome can exist in [29]. In addition, the second X chromosome in females masks male-benefiting alleles, as in half of female cells the X chromosome containing the deleterious allele will have been inactivated [29]. In agreement with data suggesting that genes involved in behavior and gonadal development are enriched on the X chromosome, more X-linked miRs are expressed in the gonad and brain than in nearly all other tissues [56].

Mammals compensate for a sex difference in X chromosome gene dosage by expressing the X-linked non-coding RNA, Xist, from a locus on one of the X chromosomes, termed the X-inactivation center. Xist spreads from this locus, and through a still relatively unknown mechanism directs the heterochromatinization and subsequent silencing of gene expression from the Xist-expressing chromosome [60]. However, this silencing is not completely penetrant. Approximately 25% of protein coding genes on the inactive X chromosome are expressed to some degree [61]. The expected result of this escape from inactivation would be female-biased expression of a large number of X chromosomal genes. Though no instances have yet been reported, the general similarities between the transcriptional regulation of protein- and miR-coding genes suggests that a portion of X chromosomal miRs likely escape inactivation as well. In fact, 86% of X-linked miRs do escape meiotic sex chromosome inactivation, a mechanism of gene dosage compensation resulting in the condensing of the sex chromosomes during and after meiotic spermatogenesis [62].

While little is known regarding X-linked miRs escaping X-inactivation in mammals, Gunaratne *et al.* report an example of biased expression of a sex-chromosome-linked miR in the auditory forebrain of zebra finches in response to birdsong, to which males and females have different behavioral responses [63]. miR-2954, located on the Z sex chromosome, displayed sex-biased expression in response to a novel birdsong. Unlike in male mammals, male zebra finches are the homogametic sex, with a ZZ sex-chromosome complement, and lack an efficient dosage compensation mechanism [64]. Accordingly, miR-2954 was expressed at greater levels in the auditory forebrain of males than females in response to exposure to a novel birdsong [63]. Interestingly, the authors identified eight predicted mRNA targets of miR-2954 that were also song-responsive, suggesting that the sex-biased response of miR-2954 may have a functional role in the sex-specific responses of zebra finches to birdsong.

**Figure 2:**
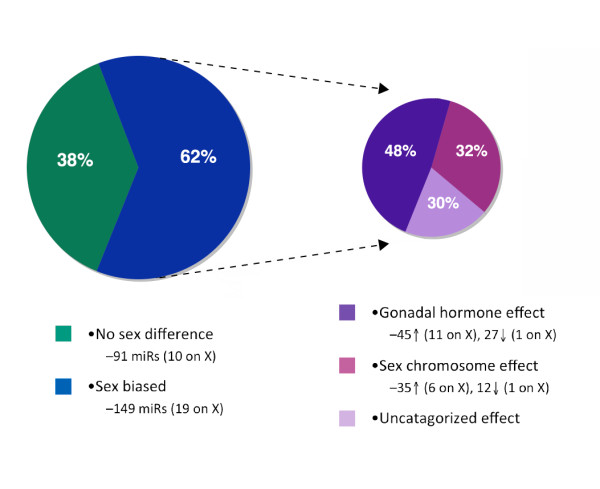
**The neonatal brain displays extensive sex bias in miR expression, which appears to result from both gonadal hormone and sex chromosomal regulation.** In our previous studies, the expression of 240 miRs was assayed in postnatal day 2 whole brains from male and female mice. To determine the role of organizational estradiol in the male brain to program the miR environment, the aromatase inhibitor, formestane, was administered to males at this time and brain tissue compared with that from control males and females. Of these 240 miRs, 149 showed sex-biased expression. These 149 miRs were then further subdivided by: 1) their apparent responsiveness to estradiol, where detected sex differences were ameliorated by formestane treatment making them regulated at some level by gonadal hormones, 2) as likely attributable to X chromosome differences where females showed higher levels (likely due to X gene dosage) and showed no changes in males treated with formestane, or 3) uncategorized effect where the pattern of change did not fit either model of gonadal hormone effect or X-linkage. Of the 149 miRs with a basal sex difference, changes related to estradiol occurred for almost half of these genes (72 miRs), where aromatase inhibition dysmasculinized male expression patterns to look more like that of the females. An effect of sex chromosomes was estimated for 47 miRs, where aromatase inhibition had no affect on male expression. Analysis criteria found that neither regulatory mechanism could be attributed to 30 miRs. (Adapted from Morgan and Bale, 2011 [11]).

In our own studies, we have identified a group of miRs with sex-biased expression in the neonatal brain that appears to result from sex-chromosome effects (Figure 2) [11]. Of 149 identified biased miRs in the neonatal mouse brain, 47 appeared to be regulated through a sex chromosome-dependent mechanism, in that the sex bias in the expression of these miRs remained even after sex differences in gonadal hormones were disrupted, though a permanent organizational effect of previous hormone differences cannot be excluded. Interestingly, only 7 of these 47 miRs were located on the X chromosome, suggesting that many of these genes may be regulated by X-linked transcription factors [11]. However, of these 7 miRs, 6 were expressed at higher levels in females, as would be expected if escaping X-inactivation. Considering these findings, it is tempting to speculate that X-linked miRs may play a particularly important role in establishing sex differences. 

### miRs regulate cell processes necessary for sexual differentiation of the brain

The first cellular mechanisms involved in sexual differentiation of the brain to be identified were those responsible for morphological differences in hypothalamic nuclei in response to estradiol during the perinatal sensitive period (as reviewed in [65]). While miRs had not been previously implicated in these specific developmental processes, work in other fields has demonstrated that miRs are important effectors in these same cellular responses in other tissues. For example, the anteroventral periventricular nucleus (AVPV) brain region is smaller in males than in females [66]. This is, in part, due to the activation of caspase-dependent apoptotic pathways by perinatal estradiol [67]. Numerous miRs have been identified in processes involved in regulating apoptosis [68]. For example, sex differences in miR-23a expression and its effect on one of its mRNA targets, X-linked inhibitor of apoptosis (XIAP), are responsible for the sex-specific activation of cell death in a model of cerebral ischemia [8]. In addition, miR-101a and miR-199a are direct regulators of COX-2, which is induced by estradiol in the developing preoptic area, and is necessary to establish sex differences in dendritic spine density in this region [69,70]. Such examples demonstrate that miRs are often regulators of processes similar to those occurring in cells undergoing sexual differentiation in the developing brain, and therefore may be the broad upstream target of estrogen’s effects during programming of the sexually dimorphic brain. Recent data from our own lab support the potent involvement of miRs in sexual differentiation of the brain at a much broader level. By examining the expression of nearly 250 of the most abundant rodent miRs, we identified a robust sex-specific pattern of miR expression in the neonatal brain where very distinct expression levels were found between males and females on postnatal day 2 [11]. Interestingly, inhibiting testosterone conversion to estradiol in male neonates at this age with the aromatase inhibitor, formestane, robustly disrupted the miR environment to the point that hierarchical clustering was no longer able to differentiate between female samples and males treated with formestane, supporting a likely involvement of miRs in estrogen’s effects [11]. It seems likely that the sex-specific pattern of miR expression we observed is a read-out of sexual differentiation at the cellular level, and that miRs play an important role in regulating these processes in sexually dimorphic neuronal populations throughout the brain. Future studies identifying the specific mRNA targets of these miRs in sexually dimorphic brain regions will likely yield new insights into novel mechanisms through which the male and female brain develops, and points of vulnerability to insults during neurodevelopment.

## Conclusions

NCBI's RefSeq project has annotated approximately 36,000 genes in both the human and mouse genome. Of these, fewer then 500, or about 1%, are on the Y chromosome and not shared by males and females [71]. Thus, the sex-biased expression of this shared genetic substrate must be an integral component of the sexual differentiation of a tissue. Evidence for this can be found in the extent to which sex differences exist in the transcriptomes of various tissues, with 55-72% of the active genes in muscle, adipose, and liver tissue displaying sex-biased expression [72]. The ability of miRs to regulate a large number of genes with a high degree of specificity and control makes them perfectly poised to play key roles in sexually dimorphic programs of gene expression. Thus, the study of miRs in sex-biased neurodevelopmental disorders, though currently lacking, could provide novel insight into pathophysiology and potential novel therapeutic targets in these diseases.

## Abbreviations

miR: MicroRNA; pri-miR: Primary microRNA; pre-miR: Precursor microRNA; RISC: RNA-induced silencing complex; NHR: Nuclear hormone receptor; ERα: Estrogen receptor alpha; ERE: Estrogen response element; AVPV: Anteroventral periventricular nucleus; XIAP: X-linked inhibitor of apoptosis.

## Competing interests

The authors declare that they have no competing interests.

## Authors’ contributions

C.M. and T.B. drafted the manuscript. Both authors read and approved the final manuscript.
